# Global brain ischemia in a dog with concurrent multiorgan dysfunction syndrome after bite wound trauma

**DOI:** 10.1186/s13028-019-0458-0

**Published:** 2019-05-06

**Authors:** Ga-Won Lee, Hee-Myung Park, Min-Hee Kang

**Affiliations:** Department of Veterinary Internal Medicine, College of Veterinary Medicine, Konkuk University, #1 Hwayang-dong, Gwang-jin-gu, Seoul, 143-701 Republic of Korea

**Keywords:** Bite wound, Dog, Global brain ischemia, Multiple organ dysfunction syndrome

## Abstract

**Background:**

Bite wounds are one of the most common traumatic injuries in dogs and depending on their severity, location, etc., urgent care including antibiotic therapy may be necessary. Serious complications can result from these injuries, such as multiple organ dysfunction syndrome (MODS), as well as a generalized reduction in cerebral perfusion, e.g. during cardiac arrest, shock, or severe hypotension that may cause global brain ischemia (GBI).

**Case presentation:**

A 5-year-old spayed female Maltese dog was presented with generalized seizures, ataxia, and obtunded mentation. The dog was injured by severe bite wounds that penetrated its abdomen and had received blood transfusions, antibiotic therapy (including metronidazole and cefazoline) and underwent emergency surgery 4 days before its visit. Based on a clinical examination, intracranial hypoxic damage with elevated intra-cranial pressure and MODS were highly suspected, and GBI was confirmed following magnetic resonance imaging. Increased signal intensity diffusely distributed in the olfactory bulb and frontal, temporal, and parietal grey matter was evident on the T2-weighted and fluid attenuated inversion recovery transverse images, along with corresponding high signal intensity observed on diffusion weighted imaging. During the 10-month follow-up period, the clinical signs gradually improved, but intermittent circling and cognitive dysfunction deficits remained.

**Conclusions:**

GBI should be included among the differential diagnoses in case of any peracute non-progressive neurological dysfunction that occurs with episodes of hypotension or hypoxia. The abnormal signal intensity observed on diffusion weighted imaging was a useful indicator for diagnosing this condition. Long-term medical management with antibiotics and anti-convulsant and anti-oxidant therapies were considered to be helpful in managing the GBI concurrent with MODS in this dog.

## Background

Bite wounds are one of the most common traumatic injuries in dogs [1]. Depending on their severity, locations, etc., urgent care including antibiotic therapy may be necessary to treat these injuries [1, 2]. Serious complications, such as sepsis, systemic inflammatory response syndrome (SIRS), and multiple organ dysfunction syndrome (MODS), may develop in both dogs and humans who have sustained extensive injuries due to dog bites [1, 2]. In dogs, a diagnosis of sepsis is established if two out of four SIRS criteria associated with infection are fulfilled [3]. MODS refers to the presence of altered organ function in an acutely ill animal [4, 5], and its pathogenesis is thought to evolve from a progressive infection that results in a non-regulated inflammatory reaction [4, 6]. Tissue hypoxia, microvascular thrombosis, increased vascular permeability, and disrupted cell–cell communication are prominent features that are associated with MODS. Supportive care, close monitoring of organ function, and intensive care nursing are needed to manage MODS [6]. The respiratory, cardiovascular, gastrointestinal, renal, hepatic, coagulation, and nervous systems are generally affected in dogs with MODS [4, 5], and multiorgan failure due to this condition is associated with mortality in both humans and dogs [4, 7, 8].

Global brain ischemia (GBI) is a multifaceted disorder that affects the entire brain after a transient period of complete ischemia, followed by reperfusion [9, 10]. A generalized reduction in cerebral perfusion, such as that which occurs during cardiac arrest, shock, or severe hypotension, may cause GBI with the hippocampus, cerebral cortex neurons, and certain basal nuclei being the most frequently affected regions [10–12].

This is the first report that describes the clinical manifestations and favorable outcome following intensive medical care administered to treat GBI in a dog with MODS.

## Case presentation

A 5-year-old spayed female Maltese dog was referred for management and diagnosis of a condition involving generalized seizures, ataxia, and obtunded mentation that developed after a surgery performed by the referring veterinarian.

Four days previously, the dog had underwent acute surgery to treat severe bite wounds that penetrated its abdomen due to an attack from another dog, which had occurred 4 h earlier. The dog was alert and had a Modified Glasgow Coma Scale (MGCS) score of 18 out of 18. The dog’s rectal temperature was 38.0 °C, and it had no signs indicating that it was affected by a systemic disease. Further, the dog received a blood transfusion, and anesthesia was induced by an intravenous (IV) administration of 0.4 mg/kg butorphanol. Moreover, the dog was given 5% dextrose in 0.9% normal saline with an IV administration of tramadol and antibiotics including cefazoline and metronidazole. However, the dog developed obtunded mentation immediately after surgery while generalized seizures and ataxia developed about 12 h later. The dog was then referred to the Konkuk University Veterinary Medical Teaching Hospital.

On presentation, the dog was obtunded and hypothermic (rectal temperature: 37.8 °C) and had bradycardia (heart rate: 88 beats/min), hypertension (systolic blood pressure: 174 mmHg), and a respiratory rate of 30/min. A neurologic examination revealed anisocoria (right > left), negative responses to menace, olfaction, a cotton ball test, hearing, and an absence of physiologic bilateral nystagmus. The neurologic findings indicated the presence of lesions in the forebrain and brainstem. The dog’s MGCS score was reduced to 11. Blood analyses revealed leukocytosis, non-regenerative anemia, azotemia, and elevated hepatobiliary enzyme levels (Table 1). The d-dimer levels and coagulation test results including the prothrombin time and activated partial thromboplastin time were within normal ranges. On an abdominal ultrasonography, increased echogenicity throughout the abdominal cavity with gall bladder sludge and heterogenous echotextures in the liver and pancreas were observed, which were indicative of peritonitis. Based on the dog’s history and results of its clinical examination, multifocal intracranial dysfunction with elevated intra-cranial pressure (ICP) and MODS were highly suspected.

**Table 1:** Complete blood count and serum biochemical results in a dog with MODS and GBI

Parameters	Day 0	Day 2	Day 3	Day 7	Day 30	Day 52	Day 114	Reference interval
WBC (10^9^/L)	18.09	25.32	25.68	37.75	9.28	8.23	8.06	5.05–16.7
RBC (10^12^/L)	4.44	3.15	5.41	4.25	6.51	7.21	7.1	5.65–8.87
HCT (%)	28.7	20.3^a^	32.6	25.6	49.2	52.1	50.1	37.3–61.7
Plt (10^3^/μL)	162	223	185	220	489	574	537	148–484
ALT (U/dL)	537	275	222	114	51	62	31	10–100
AST (U/dL)	528	302	273	172	25	37	26	0–50
ALP (U/dL)	567	452	476	393	155	222	112	23–212
GGT (U/dL)	7	–	34	74	13	9	5	0–7
Lipase (U/L)	1175	–	2299	881	714	980	1169	200–1800
CRP (mg/L)	65	50	43	39	–	29	< 5	0–35

*ALP* alkaline phosphatase, *ALT* alanine transaminase, *AST* aspartate transaminase, *CRP* C-reactive protein, *D* days after first examination, *GGT* gamma-glutamyl transferase, *HCT* hematocrit, *Plt* platelet, *RBC* red blood cells, *WBC* white blood cells

^a^The dog received a blood transfusion

Conservative treatment was initiated to control the ICP and seizures and to correct the MODS. Mannitol therapy (1 g/kg IV over 30 min; Daihan, Seoul, Republic of Korea) was started to decrease the ICP. Zonisamide (10 mg/kg PO q12h; Dapharm, Seoul, Republic of Korea) and potassium bromide (KBr; Samchun pure chemical co., Seoul, Republic of Korea) were prescribed in order to control the seizures. The loading dose of KBr was 100 mg/kg to be administered orally 4 times a day, following which a maintenance dose of 40 mg/kg was orally administered once a day. The dog continued to have 1–2 generalized tonic–clonic seizures daily for the next 2 days. Subsequently, antibiotic therapy was initiated that included the administrations of cefazolin (30 mg/kg IV q12h; Korus pharm, Republic of Korea), enrofloxacin (5 mg/kg SC q12h; Bayer, German), and metronidazole (15 mg/kg IV q12h; Daihan, Seoul, Republic of Korea) for the prevention of possible secondary infections. On day 2, following hospitalization, the dog’s anemia noticeably worsened, and it received a blood transfusion. Three days after hospitalization, the seizures occurred less than once every 24 h, and the heart rate and blood pressure normalized. The pupil size returned to normal bilaterally. The mental status of the dog improved, and physiologic nystagmus returned to normal. However, intermittent compulsive pacing and tight circling to the left side was detected, and the dog remained non-visual.

**Fig. 1:**
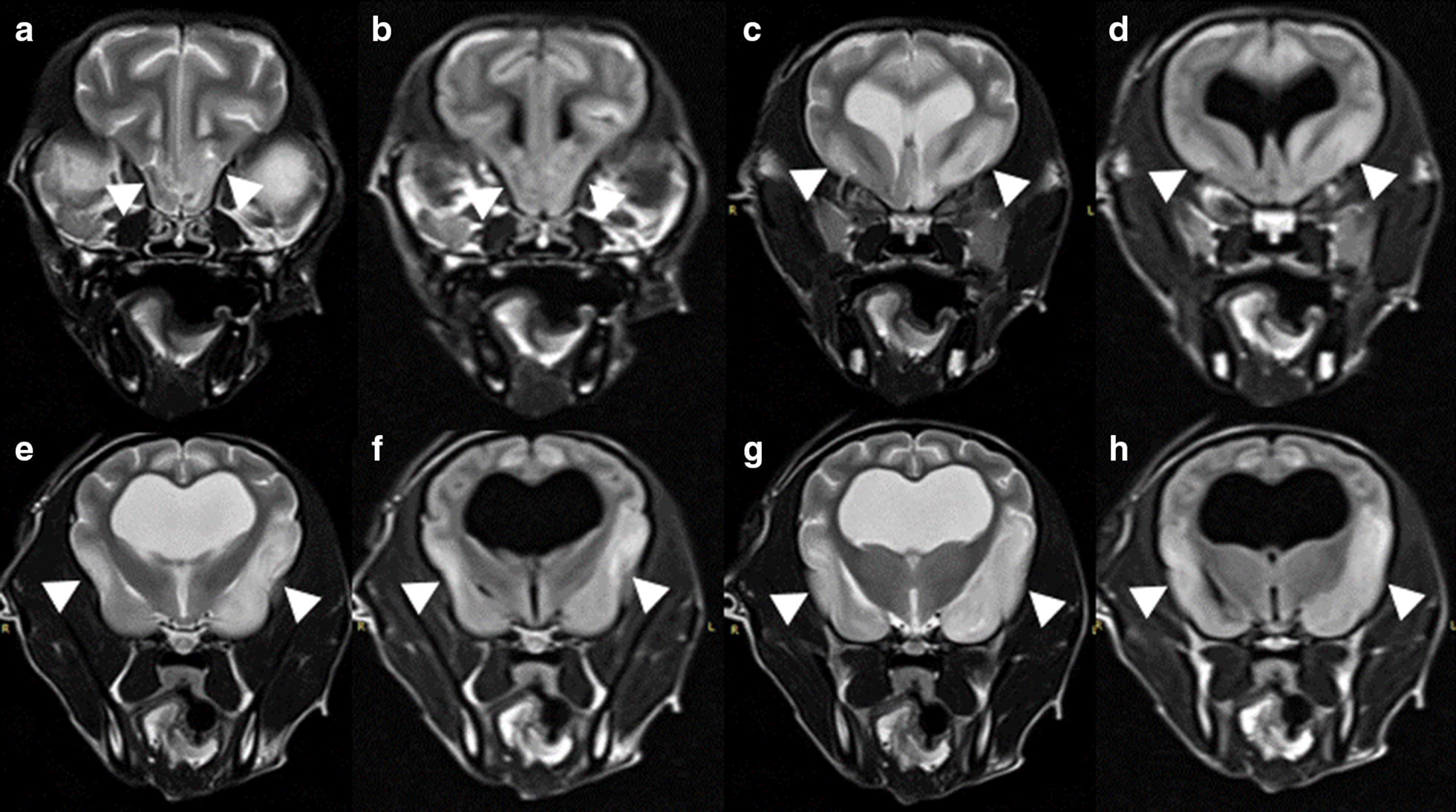
MRI of the brain in a dog with GBI. MRI showing transverse T2 (**a**, **c**, **e** and **g**) and corresponding FLAIR images (**b**, **d**, **f** and **h**) obtained 9 days after admission in a dog with suspected GBI. Bilateral asymmetric non-distinct hyperintense lesion (arrow heads) in the olfactory peduncle, frontal, temporal and parietal grey matter were observed. There is hyperintensity in these areas reflecting the parenchymal changes that occurred following the ischemic event. **a**, **b** Level of the olfactory peduncle and frontal lobe; **c**, **d** level of the caudate nucleus; **e**, **f** level of the thalamus; **g**, **h** level of the interthalamic adhesion)

**Fig. 2:**
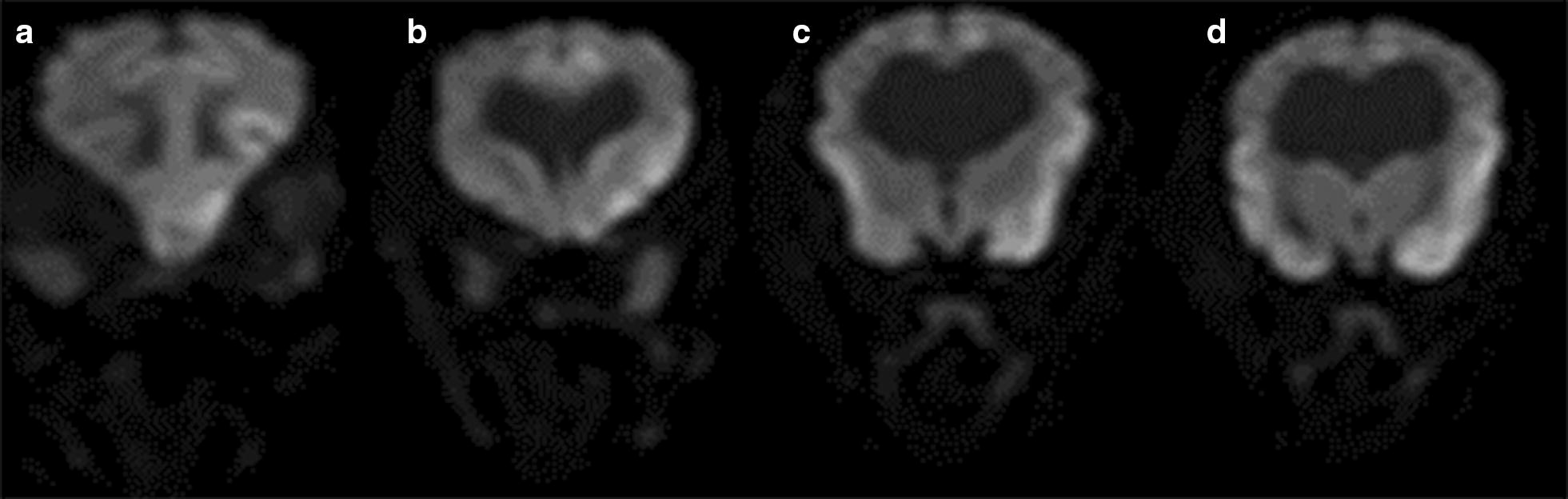
Transverse DWI of the brain in a dog with GBI. The corresponding DWI images show hyperintense lesions in the same areas of Fig. 1 but the extent and severity of hypoxic injuries are much less evident than the T2 and FLAIR images shown in Fig. 1. The lesions are hyperintense on DWI, but isointense on the apparent diffusion coefficient map, compatible with subacute ischemia. **a** Level of the olfactory peduncle and frontal lobe; **b** level of the caudate nucleus; **c** level of the thalamus; **d** level of the interthalamic adhesion)

**Fig. 3:**
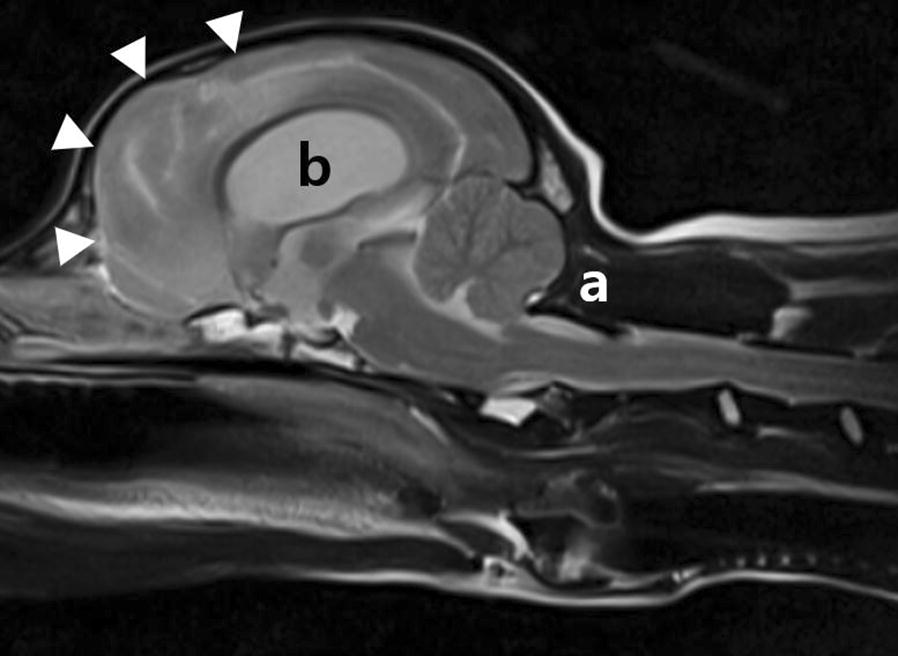
Mid-sagittal T2-weighted MRI of the brain in a dog with COMS. Cerebellar compression and herniation (**a**) and ventriculomegaly (**b**) are shown. There is hyperintensity in frontal and parietal lobes (arrow heads)

On the ninth day of hospitalization, the dog was still affected by blindness, impaired olfaction and hearing, and left-side circling. The dog showed a decreased ability to perform learned tasks, alterations in the sleep–wake cycles, and a decreased interest in food/treats and self-care (hygiene), all of which is consistent with a diffused forebrain disease. Magnetic resonance imaging (MRI) of the brain using a 1.5-T scanner (Magnetom essenza; Siemens, Erlangen, Germany) was performed to examine the intracranial parenchyma. There was evidence of asymmetrical diffusely increased signal intensity affecting the olfactory bulb and the frontal, temporal, and parietal grey matter bilaterally on T2-weighted and fluid attenuated inversion recovery (FLAIR) transverse images (Fig. 1). The differential diagnoses for the brain lesions included edema, hemorrhagic or ischemic changes. Via hyperintense diffusion weighted imaging (DWI), an isointense apparent diffusion coefficient was observed in the same neuroanatomic areas described above, which indicated the presence of subacute cytotoxic edema that was suspected to have resulted from global ischemia (Fig. 2). These findings were most consistent with GBI. In addition, severe hydrocephalus and caudal occipital malformation syndrome (COMS) were observed, which were thought to be pre-existing conditions (Fig. 3). A cerebrospinal fluid sample was aseptically collected from the cerebellomedullary cistern, and the corresponding test results were unremarkable.

Following the MRI assessment, furosemide (1 mg/kg PO q12h; Handok, Seoul, Republic of Korea) and omeprazole (0.7 mg/kg PO q12h; SK Chemicals, Seoul, Republic of Korea) were added to the previous prescription to treat the hydrocephalus. Additionally, antioxidant therapy was prescribed involving the administration of vitamin E (400 IU/dog PO q24h; Yuhan, Seoul, Republic of Korea), N-acetylcysteine (20 mg/kg PO q12h; Wooridulpharm, Seoul, Republic of Korea), and pentoxifylline (10 mg/kg PO q12 h; Handok, Seoul, Republic of Korea). The general condition of the dog improved gradually over the 10-month follow-up period. The impairments in its olfaction and hearing and the blindness disappeared, but intermittent circling to the left and cognitive dysfunction related symptoms, such as an inability to recognize the owner and to recollect learned behaviors, persisted.

## Discussion and conclusions

GBI resulting from a global reduction in cerebral blood flow causes brain parenchymal damage and neurological signs [9]. A definitive diagnosis of GBI is difficult to determine and requires investigations into the medical history and clinical signs, followed by imaging with MRI [11, 12]. Interpreting MRI findings associated with cerebrovascular lesions is complex and depends on the nature of the infarction (ischemic or hemorrhagic) and the corresponding time of onset relative to when the imaging is performed [11]. MRI is more widely available than computed tomography and is a very sensitive tool for detecting and classifying ischemic or hemorrhagic infarctions [12–14]. DWI aids in detecting early cytotoxic edema and may provide insight into the distribution of a brain injury in its early stages and the resulting mechanisms that it initiates. DWI hyperintensity, when observed throughout the entire cerebral cortex, suggests the presence of devastating diffuse hypoxic–ischemic necrosis [13, 15, 16].

GBI results from transient low cerebral blood flow and could have a variable etiopathological background. Severe systemic hypotension in the case of hemodynamic shock or cardio-respiratory arrest caused due to the reduction in oxygen concentration, decreased or abnormal hemoglobin, stroke, shock, and metabolic intoxication are the causes responsible for the development of GBI in humans [11, 19]. Experiencing an anesthetic episode is the only cause of GBI that has been reported in dogs [9, 12, 14]. In this case, the dog had a history involving hemorrhagic anemia caused due to a bite wound and an anesthetic episode induced for emergency surgery. As the dog did not have a history involving any neurological signs that manifested before the anesthetic episode, the results of the MRI findings and the clinical presentation led to a presumptive diagnosis of GBI induced by the anemia and the anesthetic episode in this dog. Multiple major clinical signs associated with intracranial dysfunction, such as blindness, ataxia, and seizures, are common in GBI patients; in this case, the dog had all of these signs, as well as apparent cognitive dysfunction that was a persistent sequela. This diagnosis could however not be confirmed by post mortem examination as the dog survived.

The brain is extremely sensitive to ischemia due to its high metabolic rate, low oxygen stores, small reserves of high-energy phosphates or carbohydrates, and few capillaries [12, 17]. Ischemic injury is less common in dogs than in humans; however, MRI findings in dogs are largely comparable to those in humans due to the similarities in the basic anatomy among large-sized gyrencephalic brains and their corresponding vascularization [18]. The precise duration that GBI requires to cause irreversible neuronal damage in humans is unknown; however, it is approximately 5 min in susceptible areas of the brain [11]. Postulated mechanisms involved in GBI include excitotoxicity, peri-infarct depolarizations, lactic acidosis, microcirculatory disturbances, and flow-metabolism uncoupling [11]. The oxygen deprivation induced by ischemia causes a switch in the mechanism of energy production from aerobic to anaerobic metabolism, resulting in the depletion of the high energy phosphate reserves, lactate accumulation, and an inability to maintain cellular homeostasis [19]. Anesthetic complications including hypotension or hypoxia can result in partial or complete brain ischemia causing temporary or permanent neurologic impairment. This can also occur in cases where no apparent complications are observed during general anesthesia as was reported in the case of a dog and a cat [9], and GBI should be considered as a possible cause responsible for any per-acute neurological dysfunction that occurs after anesthesia [12]. Although the pathophysiologies of intraoperative and postoperative brain ischemic events that develop after anesthesia remain uncertain, significant neurological recovery is possible [12]. The MGCS score of the dog was 11 at presentation, indicating a poor to guarded prognosis [20]; however, the score did not decrease after discharge; on the other hand, it improved.

In this case, severe hydrocephalus and COMS were observed to be concurrent with GBI. Hydrocephalus can be classified as either being congenital in origin or having been acquired clinically. Congenital hydrocephalus is more common than the acquired type and is most common in toy breed dogs, such as Maltese, English bulldog, Pug, Pomeranian, Yorkshire terrier, and Chihuahua [21, 22]. In addition, congenital hydrocephalus may be concurrent with other nervous system anomalies, such as COMS and meningomyelocele [22]. In dogs, acquired hydrocephalus can develop at any age secondary to trauma, tumors, and meningoencephalitis leading to obstructions in the ventricular system [21]. In humans, the incidence rate of ventricular dilation occurring after a severe traumatic head injury is known to be about 39–44%, and increased ventricular size was found to be evident 4 weeks after sustaining an injury [23]. One report showed that the prevalence of hydrocephalus among dogs with head trauma was 26% (7/27), which was concluded to be an incidental finding [24]. There is no report on acquired hydrocephalus following the development of GBI in both humans and dogs. Considering the dog’s breed and that it was affected by a COMS and pre-existing ventriculomegaly without delay of onset, it is likely that the dog has congenital hydrocephalus rather than acquired hydrocephalus.

Dogs can be diagnosed with sepsis if at least two out of four SIRS criteria associated with infection are satisfied [3]. Hardie’s SIRS criteria are as follows: body temperature < 38 °C or > 40 °C, heart rate > 120 bpm, respiratory rate > 20/min, white blood cells (× 10^3^/μL) < 5 or > 18, or band cells > 10% [3]. MODS is continuous and gradual dysfunction of organ function in an acutely ill animals, and the various organ systems are generally affected [4, 5]. In this case, the dog satisfied Hardie’s SIRS criteria, and hepatic and neurologic dysfunctions were found at presentation with suspected peritonitis, indicative of MODS.

In this case, the diagnoses of GBI and MODS were made based on the dog’s history, clinical signs, and MRI findings. Although the dog had a severe complicated illness concomitant with MODS and GBI, these diseases were managed successfully with anticonvulsants and antioxidant therapy. To the author’s knowledge, this is the first report on the diagnostic features and clinical outcomes of GBI concurrent with MODS in a critically ill dog after it sustained bite wound trauma.

## Data Availability

All data generated or analysed during this study are included in this published article.

## Abbreviations

COMS: caudal occipital malformation syndrome
DWI: diffusion weighted imaging
FLAIR: fluid attenuated inversion recovery
GBI: global brain ischemia
ICP: intra-cranial pressure
KBr: potassium bromide
MGCS: Modified Glasgow Coma Scale
MODS: multiorgan dysfunction syndrome
MRI: magnetic resonance imaging
SIRS: systemic inflammatory response syndrome
